# Agribusiness, Corporate Social Responsibility, and Health of Agricultural Migrant Workers

**DOI:** 10.3389/fpubh.2016.00054

**Published:** 2016-03-29

**Authors:** María Isabel Ortega, Samantha Sabo, Patricia Aranda Gallegos, Jill Eileen Guernsey De Zapien, Antonio Zapien, Gloria Elena Portillo Abril, Cecilia Rosales

**Affiliations:** ^1^Coordinación de Nutrición, Centro de Investigación en Alimentación y Desarrollo, A.C., Hermosillo, Mexico; ^2^Mel and Enid Zuckerman College of Public Health, The University of Arizona, Tucson, AZ, USA; ^3^Centro de Estudios en Salud, El Colegio de Sonora, Hermosillo, Mexico

**Keywords:** corporate social responsibility, agribusiness, farmworkers, migration, Mexico

## Abstract

**Background:**

Living conditions and health of migrant farmworkers could benefit from a health promotion model based on corporate social responsibility (CSR).

**Objective:**

To understand how Mexican agribusiness owners and general managers view and practice CSR.

**Methods:**

We interviewed 8 agribusiness owners/managers and 233 farmworkers using open-ended interviews and gathered anthropometrical data of 133 children from farmworkers families. To guide our analysis and discussion, we followed the two-dimension model of CSR proposed by Quazi and O’Brien.

**Results:**

According to interviewee responses, mean percentage of agreement with CSR concept was 77.4%, with a range of 54–85.7%. Main health-related issues among farmworkers were infectious diseases, crowding, and access to health-care services; there were acute cases of undernutrition among farmworkers’ children and diets were of poor quality.

**Discussion:**

Agribusiness owners and managers understand and practice CSR according to a wide and modern view, which contradicts with farmworkers’ living conditions and health. Quazi and O’Brien model should consider the social context, in which it is analyzed, and the social manifestations of community development as a tool for further analysis on the perceptions and actions of entrepreneurs.

## Introduction

Mexico’s migrant farmworkers are among the poorest and most vulnerable populations in the country. Economic vulnerability in this population includes cultural and linguistic marginalization, as more than one-third of Mexican nationals (39.4%) are indigenous and half are monolingual non-Spanish speakers (1, 2). According to the national farmworker survey (2), there are 2,040,414 farmworkers within Mexico with an average family size of 4.5; from which 762,265 are migrant farmworkers. This national survey also estimates that the average age of migrant farmworkers is 36.3 ± 14.2 years.

Intergenerational poverty perpetuates the process of internal migration to urban centers, to the richest states in the country, and for many, to the United States, as undocumented farmworkers. Families of migrant farmworkers experience higher poverty rates than the general population, thereby increasing the synergism between poor nutrition and a depressed immune system (3). This scenario and the living conditions of communities or agricultural fields, where they arrive, expose the individual to both infectious and chronic degenerative diseases (4).

Sonora located in northwest Mexico has the second highest number of farmworkers in the country. Each year, it is estimated that between 100,000 and 150,000 workers are hired in the Sonora agribusiness, of which 80,000 are considered migrants. The annual mass migration drains resources from the farming communities of Sonora. Towns of less than 1,000 people grow near to 60,000 during the busiest work season (2, 5). This demand for resources is felt in various social and environmental aspects, especially in the provision of adequate primary health care. In 1997, the Mexican Social Security Institute (6), the country’s main public health provider, extended its coverage for temporary farmworkers; this, however, does not mean access to all employment benefits as year round workers. Employers and intermediaries or contractors are required to register and contribute financially to the welfare of their workers, but often do not (7). The states of Sinaloa and Sonora of northern Mexico have been the most successful and are leaders in hiring migrant farmworkers. However, throughout Mexico, including the state of Sonora, companies often submit incomplete lists of workers and do not inform their employees of their right to register with IMSS (8, 9).

In addition, living conditions and health of migrant farmworkers in the state of Sonora face challenges associated with poverty in the community and the living conditions and environmental factors in agricultural fields where they work and live. The discussion of these living conditions and health issues has been published previously (4, 10, 11) and include overcrowding in farmworker housing and barracks, which facilitates the transmission of respiratory infectious diseases; inadequate availability of health services and areas of personal hygiene, feeding practices inconsistent with the nutritional needs of a worker with high energy expenditure, and the lack of food safety practices to ensure the health of workers. Also, the conditions of health and nutrition of children in migrant farmworkers’ families in Sonora and Baja California in northern Mexico indicate that stunting and underweight are higher than the national average, which predicts future inadequate health development if poverty conditions continue (11). Health care is further complicated by the large distances, i.e., workers have to travel to get to clinics that offer public health services, the high cost of services and drugs, and the low percentage of enrollment in public Social Security, which is the system of health care designated for seasonal migrant farmworker population (6). The health and nutritional status of farmworkers and their families is a priority and requires monitoring and systematic attention because it not only ensures family sustainability but also influences productivity, from the perspective of the agribusiness a healthy worker is also a more productive worker (4, 10).

As consumers from developed countries become increasingly more and more aware of social injustices in the agricultural business, exporting companies from countries such as Mexico growing progressively are aware of the benefits that attention to the living conditions of workers brings to their businesses. It is not only recognition of their social responsibility but also ensures food safety, especially in perishables (12).

### Corporate Social Responsibility

Corporate social responsibility (CSR) can be summarized as “*the voluntary behavior of companies to go beyond the legal requirements of the country in which they operate, given their long-term interests for integrating economic, social and environmental impacts to their operations*” (13). Essentially, CSR is considered a comprehensive business model, not just another basic activity of the company. CSR is how “everyday business” should be conducted in a global economy. The European Commission (EC), which provides one of the most recognized definitions of CSR, promotes and emphasizes the integration of social and environmental concerns in the strategy and operation of businesses. The EC also suggests the importance of how businesses interact with their internal and external partners (employees, customers, neighbors, NGOs, authorities, etc.) (13).

The evolution of schools of thought associated with the concept of CSR parallels the evolution of the economic system and industrial transformation that societies have been through in the last century. Some authors have questioned whether CSR really has any impact on the welfare of society (14). Such discourse is focused in three major schools of thought: business ethics, business and society, and social aspects of management (15). The first area of business ethics describes the moral responsibility, which that a company should behave socially responsible “because it is the right thing to do”; on the other hand, the concept of business and society is based on the assumption that business and society are part of the same system and are in constant interaction and that they are bound by a social contract; therefore, companies are subject to the control of society. Thus, companies are created to meet a certain role in society and their legitimacy depends on how well they meet. The third school of thought, social aspects of management, represents a utilitarian purpose; social problems are part of a strategic management and are based on market opportunities created by the change in social values. If business can anticipate and respond to these phenomena with advantage, socially responsible behavior can generate a competitive edge and a company can proactively anticipate the impending regulations or can even avoid them (16).

Other researchers have generated conceptual models, which describe a continuum of CSR, and have identified that business typically fall in two primary categories, including a reduced or broad vision of CSR. A reduced or classical vision of CSR assumes CSR as predominantly economic or legal. Corporations are considered legal entities with two fundamental responsibilities: to generate profit for the owners and their partners and to comply with the law. A broad or modern vision posits these basic principles and moves beyond them to include moral, ethical, and philanthropic responsibilities (17–19). Furthermore, a modern view recognizes that businesses are managed by individuals or ordinary citizens, and as members of society, these citizens have an obligation to comply with the principles of morality, responsibility, and integrity. Inherent in this view, is the business owner’s obligation to assume the liability associated with meeting a broader spectrum of CSR; including protecting the environment, conserving natural resources, participating in community development, and conducting philanthropic donations. Those who adopt the modern view, consider CSR as an umbrella term for various theories and practices that recognize: (1) corporations are responsible for their impact on society and the environment, sometimes beyond legal compliance and responsibility of individuals; (2) companies are responsible for the behavior of others with whom they do business (e.g., suppliers in the supply chain); and (3) companies need to manage their relationship with society in general, whether for reasons of commercial viability or to contribute to society (19, 20).

Corporate social responsibility is of growing interest in the globalized marketplace, especially in the areas of agribusiness and the food industry, however, within these industries the CSR definition and scope remains a challenge highly dependent on the view, be if classical or modern, from which it is operationalized (21, 22). CSR models, regulations, and voluntary standard adoption in agriculture are 5–10 years beyond that of the industry, and thus, require empirical data to explain the wide variety of standard development and adoption within the unique context of agriculture (22). Given the lack of CSR models generated and or applied within the context of agribusiness, we draw on Quazi and O’Brien’s two-dimensional model of CSR. This model developed and validated the constructs and measurements to assess corporate leadership perspectives of CSR. Compared to other models, Quazi and O’Brien enabled the analytical power to understand the complex phenomena of CSR and to identify the inconsistencies between belief and application of the principals of model CSR. Furthermore, these researchers developed their model within the context of multinational corporations operating in the developing world context – which was important to our work as our study took place in Mexico – classified as a middle income country.

This article seeks to understand and address health and social issues that migrant farmworkers in northern Mexico and the southern United States face, from the perspective of migrant farmworkers employed as seasonal workers in large Mexican agribusiness and the agribusiness employers who hire them. Specifically, we aim to assess the theoretical position of the owners of agribusinesses on CSR and compare this perceived position with health and living conditions experienced by the migrant seasonal farmworkers and their children employed in their agribusiness.

## Methodology

Data were collected through the collaborative work of the Research Center for Food and Development, A.C. (CIAD, A.C.) (Centro de Investigacio’n en Alimentacio’n y Desarrollo, A.C.), the College of Sonora (COLSON) (El Colegio de Sonora), and the University of Arizona, Mel and Enid Zuckerman College of Public Health. In 2007, we formed a binational interdisciplinary work group to focus on health issues facing the migrant farmworker population in the north of Mexico. Our binational team set out to build on 5 years of previous migration and health research conducted by CIAD. We developed a cross-sectional pilot study to understand models and public policy issues related to CSR in agribusiness and the responses of agribusiness to these models and explored the challenges of migrant farmworkers in accessing adequate living and working conditions as well as health-care services. Each of the three components of the original project plan followed a specific methodology included in the collection and analysis sections.

### Survey Development and Sampling

#### Agribusiness Owners

In the area of *San Miguel de Horcasitas (Pesqueira), Guaymas and La Costa of Hermosillo*, located within the state of Sonora at northwestern Mexico, there were approximately 200 companies producing mainly grapes and vegetables during the period of study. The study population consists of approximately 120 of those agribusiness representatives involved in the production of table grapes and/or fresh produce (vegetables) in the region. Sampling focused on export-oriented agribusinesses in the state of Sonora dependent on a high volume of migrant seasonal agricultural farm labor (farmworkers). The agricultural growers were identified from a list generated by the association of producers in the region and the governmental program that oversees the migrant farmworkers Fundación Produce, a public–private participation, and PRONJAG, the National Program with Agricultural Laborers, under the Ministry of Social Development (SEDESOL).

**Figure 1:**
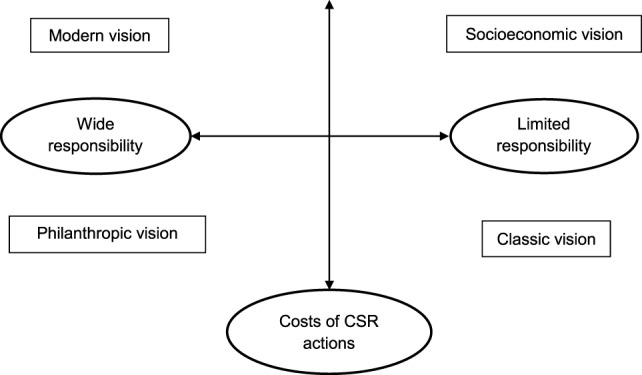
**Two-dimension model of CSR (18)**.

A team of researchers approached the owners, first through presentations of the goals of the study to Fundación Produce and the PRONJAG meetings attended by potential participants. Interested participants were then contacted *via* phone and e-mail and invited into the study. A face-to-face structured interview was later scheduled in a convenient location. A total of eight owners or general managers of agricultural enterprises (three grape and five vegetable growers) were interviewed using a structured format with open- and closed-ended questions. The interviews were conducted by members of the multidisciplinary working group, which had established a relationship with the owners and managers of farms through prior action research projects (7). The survey was developed by a multidisciplinary team over a period of time and consisted of four sections: business profile, company overview, labor market and welfare of workers, and certification and CSR. The two-dimensional CSR model proposed by Quazi and O’Brien (18) (Figure 1) was the theoretical guide. This model guides the conceptualization of research tools, analysis, and perception of CSR among owners and managers of Sonora’s agribusinesses. Thus, to identify the theoretical position of the owners of agribusinesses about their perceptions on costs and benefits of engaging their business in social responsibility activities, we adapted the Quazi and O’Brien instrument to include a 14-item, 1–4 likert scale that covered a wide range of social responsibility issues. The wording of these questions follows the format used by Quazi and O’Brien (18). The questions were developed to encourage the respondent to answer the level at which they agreed or did not agree.

#### Migrant Farmworkers

Health and living conditions of farmworkers was assessed through face-to-face surveys with 233 migrant farmworkers employed in four of the largest agribusinesses in Sonora. Interviews were conducted during the height of the growing season between April and November of 2007. The interview questions were derived from instruments developed in the United States, including the National Agricultural Workers Survey, the California Agricultural Workers Health Study (23), and the Binational Farmworker Health Survey (24) from instruments developed in Mexico by the Mexican Secretariat of Social Development (SEDESOL) and the National Agricultural Farmworker Program (25). The questions addressed aspects, such as demographics, working and living conditions, health status, health services and governmental aid program access, as well as training regarding personal hygiene, pesticide management, and food safety. Workers were approached to participate in interviews during packing in the fields, rest periods, and after work. The interviews, which were conducted with individuals, lasted between 15 and 30 min. Furthermore, in order to document the impact of living conditions within the farm, the study also collected dietary interviews (24-h recall) with 63 adult men and women and analyzed 9 menu registries from 2 of the farms’ dining halls. Based on the Dietary Guidelines for Americans (26), we categorized food consumption by type and nutritional content, in order to have a qualitative examination of diet adequacy. Descriptive statistics were used to analyze data from individual interviews.

#### Migrant Farmworker Children

Anthropometric data was measured from 133 children with a mean age of 28 months. We measured weight, height, and age to compose nutritional indicators of weight/age, height/age, and weight/height (27).

### Human Subject Committee Approval

This study was approved by the Human Subjects Protection Program from The University of Arizona on February 13, 2007.

### Data Analysis

The data collected from owners/managers interviews, as well as data from farmworkers interviews, was analyzed by descriptive statistics and the SPSS statistical package, version 14 (28). Short answer narratives with farmworkers were also transcribed and loaded into NVivo qualitative data management software (29). All interviews were independently reviewed by two researchers and through a face-to-face discussion – researchers came to consensus on the meaning, intent, and context of qualitative responses. These data were then brought to the full team for interpretation. For purposes of this paper, we include quotes that illustrate and provide meaning to the quantitative findings. A dietary database, including data from the USDA (30) and CIAD on regional foods, was used to analyze the nutrient supply of farmworkers’ diets (30, 31).

## Results

### Characteristics of Agribusiness

A total of eight owners or general managers of agricultural enterprises (three grape and five vegetable growers) were interviewed. The average age of participants (businessmen owner/grower and managers) was 54 years with a range of 39–69. Seventy percent obtained a professional education in agriculture and/or agribusiness administration. On average, they have been involved in this activity 22 years, ranging from 12 to 58 years. In the case of grape production, the average number of years in this business was 18 and for vegetables 34 years. For those engaged in table grapes or vegetable production, 100 and 20%, respectively, indicated that agriculture is not their only business. These entrepreneurs have diverse business portfolios that also include poultry farming, fishing, real estate, and banking. To maintain the fields of table grapes active all year, vegetables are also produced. Eighty percent of vegetable growers surveyed indicated that they devote 100% of their time to this activity.

Regarding the agricultural area of the production units (APU), table grapes are grown in an area that, on average, covers 460 ha and vegetables 401 ha, within a range of 50–1,000 ha. The sample includes one APU, which is significantly greater (10×) than the rest of the sample remaining area (Table 1). Production of both crops was slated for export and, therefore, is subject to the requirements of a globalized market. In both types of crops, produce is sent mainly to the United States (73%) and to a lesser degree Canada (7.6%). A smaller amount of grape production and vegetables, 7.3 and 23.6%, respectively, supply the national market in Mexico. With this amount, the producers have a presence in the domestic market, expecting it could grow in the future. It also serves as a buffer for unforeseen fluctuations in the international market. Per special request, one of the producers of vegetables sends 15% of its production to Europe.

**Table 1:** **Agricultural production units characteristics (APU, *n* = 8)**.

	Farm type	
	Grape	Vegetables
Cultivated area (Has.)	460	401^a^
Production destination (%)
National market	7.3	23.6
USA	73	73.4
Canada	7.6	7.6
Europe	6.6	15.0^b^
Average number of workers
Seasonal	1700	370
Permanent	93	98
Associated enterprise (%)	0	60
Member of a produce association (%)	100	100
Member of an exporting association (%)	33	0

*^a^APU is significantly larger than the others*.

*^b^APU significantly larger than others, by special request*.

The number of farmworkers by APU and the existence of business partners in the ownership of the company differentiated producers of table grapes from vegetable producers. In the case of grapes, on average 370 season farmworkers were hired and 1,700 hired for vegetables. In regards to partners in the business, 100% of grape growers declared not having business partners. In contrast, 60% of vegetable growers rely on business partners.

A common feature in both types of agricultural crops is that 100% of its ownership belongs to at least one association of producers; however, for exporting, responses indicated that they are not part of an organization. In some cases, the Grape Growers Association has an active role in this process. With respect to the requirements or certifications necessary to market their produce, responses in both types of crops are clear: the domestic market is not as demanding but benefits indirectly from those required by international markets (Table 2). Clearly, in their farming practices, they meet the requirements asked by the importing countries or companies that buy produce. Almost all the requirements referred to are included under plant health or “Good Agricultural Practices” (good water management, fertilizers, fungicides, pesticides, training workers, etc.)

**Table 2:** **Global markets certification requirements**.

Country or region	Required certification
Mexico	Servicio Nacional de Sanidad Inocuidad y Calidad Agroalimentaria (SENASICA)
USA	US Department of Agriculture (USDA)
	Food and Drug Administration (FDA)
	Hazard Analysis and Critical Control Points (HACCO-USDA/FDA)
	Primus Laboratories
	Servicio Nacional de Sanidad Inocuidad y Calidad Agroalimentaria (SENASICA)
Canada	“Same as with North America”
European common market	Euro-Retailer Produce Working Group-Good Agricultural Practices (EureGAP)
	Natural choice
	Field to fork
	Kosher
	Servicio Nacional de Sanidad Inocuidad y Calidad Agroalimentaria (SENASICA)

*Source: own study data 2,000*.

### Corporate Social Responsibility

According to the quantitative assessment adapted from Quazi and O’Brien (18) to identify the two main classifications of CSR, which include the broad and the narrow or classical views, participating agricultural entrepreneurs can be characterized as embracing a broad view of CSR (Table 3). Responses to the 14-item questionnaire clearly demonstrate that owners and managers agree that business are part of a larger society and should therefore respond to social issues (A4) and that by contributing to such social problems one can make a profit (A6). Corporate social action programs were believed to contribute to a favorable image for their business (A15) and that regulation was not sufficient in ensuring that business behaved in a socially responsible way. Participants clearly disagreed with factors associated with a classical or narrower view of CSR. Specifically, participants agreed it was fair and necessary to require businesses to contribute to social programs. This broad view was further demonstrated in the score of the theoretical agreement with the concept of CSR (Table 4). The rating is based on a range of 0–100. Agricultural businessmen with a score near 100 show a consistent theoretical agreement with the various elements of the concept of CSR and show a greater willingness to implement these concepts in daily operations. The average rating was 77.4%, with a range of 54–85.7%.

**Table 3:** **Mode of responses of owners/managers to CSR-related items (*n* = 8)**.

Questionnaire items	Mode
• Business should realize that it is a part of the larger society, therefore it should respond to social issues	5
• Social regulation has already put a check on business behavior and it is unnecessary for business to be involved in social responsibility programs	2
• Contributing to the solutions of social problems can be profitable to business	5
• Regulation is not sufficient to ensure business behaves in a socially responsible way	4
• Business should tackle only those social problems that are created by its own actions	2
• Business already has a lot to do and should not take on other responsibilities	2
• Society expects business to help solve social problems as well as to produce goods and services	4
• Business is primarily an economic institution and it is most socially responsible when it attends strictly to its economic interests	2
• Corporate social actions programs can help build a favorable image for a business	5
• Business has a definite responsibility to society apart from making a profit	5
• Business that ignores social responsibility may have a cost advantage over a business that does not	4
• It is unfair to ask business to be involved in social responsibility programs as it is already doing to by complying with social regulations	2
• Society expects business to contribute to economic growth as its only concern	2
• It is unwise to ask business to fix social problems created by others and which have not profit potential	2

*Item response: 5 = totally agree, 1 = totally disagree*.

**Table 4:** **Level of theoretical concordance with Quazi and O’Brien CSR concept (*n* = 8)**.

Agribusiness owner or manager	Total (%)
A	59 (84.2)
B	59 (84.2)
C	60 (85.7)
D	57 (81.4)
E	53 (75.7)
F	46 (65.7)
G	50 (71.4)
H	50 (71.4)
Average	54.2 (77.5)
Range	54–85.7
Total possible	70 (100)

Producers were asked a series of questions regarding their definition and application of CSR (Table 5). None of the producers could provide an adequate definition but were able to give examples of federal and state programs that assist farmworkers families in health and social services. When probed about how they apply aspects of CSR, responses became much more concrete. Motivations to apply concepts or standards of CSR derived from a personal desire to improve the lives of their employees, principally farmworkers, and their families. Vegetable producers connected CSR with occupational health and safety and injury prevention. Both types of producers view United States-based corporations, such as Wal-Mart and Costco, as agents promoting CSR. The majority of responses named CSR actions of environmental protection efforts to reduce toxic residues and “attention to workers” as key examples of how companies with whom producers trade promote CSR. Producers were also asked to name the types of certifications required by the international (and national markets) in which they interact. Most responded to this question with food safety requirements required by the United States Food and Drug Administration or the United States Department of Agriculture. The producers exporting to the European Union listed certifications that moved beyond the traditional and albeit important food safety protocols to include Global G.A.P, Field to Fork, and Kosher, all of which require some element of protection of worker aspect (Table 5).

**Table 5:** **Agribusiness vision of corporate social responsibility (CSR)**.

	Type of crop	
Agribusiness owners and managers responses (*n* = 8)	Grape	Vegetables
CSR definition	SEDESOL programs	CIAD programs
	ALTA foundation programs	Ministry of economics programs
		Alianza Para el Campo programs
CSR implementation	Health programs	Adequate salary
	Nutrition Children’s education	Human rights Training Employment protection
Motivation to implement CSR	Assure workers improvement	Improve workers’ living conditions
	Improve workers’ living conditions	Benefits for workers and farms Less lost work days
CSR promoting business	Costco	Costco
	Wal-Mart	Wal-Mart
CSR promoting business issues	Workers health care	Workers health care
	Environmental care Diminishing toxic residues	Diminishing toxic residues

*Source: own study data*.

### Migrant Farmworker Health

#### Demographics and Living Conditions

Among the 233 migrant farmworkers interview, 69% were employed in grape producing farms and 30.9% in vegetable producing farms. Forty percent were women and 60% men. Most of farmworkers were from indigenous origin (Tzotziles, Tzetzales, and Náhuatl) and came from the southern states of Chiapas, Veracruz, Puebla, and Guerrero; although there were some farmworkers from the northeastern states of Sinaloa and Sonora. Minimum age of interviewed farmworkers was 18 years and maximum was 72 years; though 80% of the sample was younger than 40 years of age. Farmworker short narratives describe the differences between farmworker hometowns, which are predominately mountainous and coastal regions of southern Mexico, compared to the agricultural regions of Northern Mexico, which are predominately arid deserts:

It is so different here, there is no work at home that is why we come to suffer here … where we live there is no piped water, no electricity, but there is a river, and lots of fruits that grow wild, not as it is here where they have to put too much garbage (fertilizers) for the (fruits) to grow well … at home the fruits grow natural

Crowding is common in farmworkers housing. Among those migrant farmworkers traveling with extended family members, approximately 42% shared a small room with 5–11 family members. Among those migrant farmworkers traveling alone, approximately 56% shared a common living and sleeping space with 30–60 individuals without privacy. Each room was fitted with three tiers of small cement beds stacked one above the other.

#### Health Status and Access to Health Services

As we can see in Table 6, during the study period, 58% of the sample reported having been sick or had some kind of body pain. The main illnesses reported were infectious diseases (70%), such as respiratory and gastrointestinal illness, related to change in weather, crowding, food, and work, including tasting grapes to ensure maturity. Thirteen percent reported lesions of skin and eyes, muscle pain, and accidents; meanwhile, 8% reported headache and symptoms of dehydration. Only 3% reported diagnosis of diabetes, cancer or hypertension. Farmworker short narratives further characterize the health status of their coworkers and family members, including children of farmworkers who are often ill with diarrhea and respiratory infections:

Children get diarrhea all the time … perhaps because the milk [that is prepared for the child at the nursery] makes them sick…that boy there, his mother had to take two days off work because her child. He is sick all the time…

Right now children are getting sick with flu. And there is no nebulizer at the farm’s clinic [clinic is owned and operated by the agribusiness] even when we need it so much. We need an apparatus like this, but they (at the clinic) do not care what it is needed.

**Table 6:** **Main health problems among migrant farmworkers from three farms in Sonora (*n* = 233)**.

	Adult men (60%)	Adult women (40%)
Reported illnesses (%)	51.6	67.9
Infectious		
Gastrointestinal	14.2	25.6
Respiratory	28.4	24.4
Other (dehydration, headache)	0.6	5.1
Diabetes, cancer, and hypertension	2 (1.3)	2 (2.6)
Anemia	0 (0)	2 (2.6)
Work injuries	6 (3.9)	2 (2.6)
Muscle and bone injuries	5 (3.2)	4 (5.1)
Pregnancy related	0 (0)	1 (1.3)

*Source: own study data*.

Three quarters of farmworkers interviewed were not enrolled in the Mexican Institute for Social Security (or IMSS), which serves as the main provider of health and social benefits of seasonal or temporary agricultural workers. In addition, those that reported having this service, commented on some access difficulties, such as distance, transportation cost, discrimination, and delays. In two of the farms, there primary health-care services were offered through a farm clinic owned and operated by the agribusiness. However, these clinics were often staffed by one medical student resident, responsible for an average of 300–1,800 workers per season. Farmworkers described the scarcity of medical providers on the worksite and the distance required traveling to be attended by a clinic in the nearest town, which are often small agricultural communities:

Here [at the farm] there is no full time doctor, the doctor just comes from 5 to 6 p.m. … and it is difficult for us to go looking for health services in town, because the bus to town only runs two or three times a day and costs 300 pesos [approximately 16 USD] This is very difficult.

When we get sick, we go to the farm clinic [owned and operated by the agribusiness], but only for fever or a mild cough. When there is a serious health problem, they [clinical staff] take us into the town health clinic or to IMSS (Mexican Social Security Institute) … but if we get sick when there is no doctor at the farm clinic then it could be bad …

#### Nutritional Status

Height for age, weight for age, and weight for height of boys and girls (Mean *z* score being −0.4525 ± 1.2, −0.3485 ± 1.2, and 0.1032 ± 1.1 years, respectively), indicate adequate physical growth, according to WHO reference standards; however, 10.5% of children showed stunting (low height for age), 8.3% showed low weight for age, and 3% showed wasting or low weight for height. Interestingly, the percentage of low weight for age (an indicator of acute malnutrition) is higher than that reported for general population of children in the same age in the state of Sonora and also compared to the prevalence of low weight for age at a national level (8.3 vs. 5.2 and 5%).

Analysis of the menus offered by agribusiness, farmworker diets were found slightly low in protein (13% of total energy), although, carbohydrates and fat intake (64 and 22%, respectively) fulfilled recommendations for an adequate health status. On the other hand, micronutrients intake revealed poor quality diets of farmworkers, since Calcium, Vitamin A, C, and E were lower than the recommendations provided by the Institute of Medicine (32). Based on our analysis, a typical diet of a farmworkers included corn tortilla, eggs, beans, rice, chicken soup, tomatoes (as condiment), pork sausages, soda pop, and coffee. Corn is usually fortified in Mexico with iron, zinc, and folic acid, which prevent deficiencies in these areas among farmworkers. It is important to clarify, however, that protein and iron in farmworkers diets was derived mainly from cereals, which make these micronutrients biologically less unavailable. Farmworkers describe their food experiences and the monotony and poor quality of the food served by the agribusiness cafeteria, which is the only option for farmworkers and their families:

At the cafeteria they offer eggs for breakfast, every day is the same, every day, there is no change in the food they offer, and dinner is not that different, so it is not very satisfying, you know…It is true that you are not expecting to have meat every day or in three meals per day… but every food should be well cooked, tasty, even if there is just beans, but well cooked, and tasty. Sometimes they serve burned beans or very fatty foods…people have stomachache, sometimes due to food.

## Discussion

Through triangulation of quantitative and qualitative data drawn from various perspectives and vantage points, including those of Mexican agribusiness owner, migrant farmworkers, and the nutritional status of migrant farmworker children, we found a distinct difference between agribusiness owners’ perspectives and perceived behavior related to CSR and the health and social realities of the migrant farmworkers they employ. Specifically we found that agribusiness owners involved in this study positioned themselves as holders of a broad or modern view of CSR. They perceived themselves as members of society with an obligation to comply with the principles of morality, responsibility, and integrity. Such a vision also suggests that they as business owners have an obligation to assume the liability associated with meeting a broader spectrum of CSR; including protecting the environment, conserving natural resources, participating in community development, which involves human development, and conducting philanthropic donations. Yet, such a modern CSR position is juxtaposed with extant evidence of deplorable living and working conditions among the migrant farmworkers they employ, including high frequency of diarrheal and upper respiratory infections, related to overcrowded living conditions, lack of access to primary health care, and low quality and unhygienic nutritional services. Specifically, even without being familiar with the definition of the concept of CSR and its theoretical principles, the agribusiness responses suggest that employers are sensitive to trends and demands of the international market. One must remember that their incursion and presence in the international market is the result of incorporation of production technology and compliance with health standards set by global markets. This could indicate that in the evolution of international markets, incorporating the concept of CSR as a production system in agricultural enterprises has a promising future. CSR could become an element that allows agribusiness entrepreneurs to not only remain competitive in the international environment but also contribute more widely in the local environment in which they work.

### Corporate Social Responsibility Operationalized

The International Labor Organization (ILO) calls for businesses to guarantee workers safe and healthy working conditions, access to basic health, education, and housing (33). Yet to compete globally in agriculture, the supply chain model encourages farm labor contractors, growers, processors, suppliers, buyers, retailers, and investors to cut production costs at every opportunity. Cost savings impact farmworkers in the form of below subsistence living wages, dehumanizing living conditions, and human and civil rights violations, ultimately affecting the health of this critical workforce. For migrant seasonal farmworkers of Mexico, the social determinants of health (SDH) include the living and working conditions that affect the opportunities farmworkers and their families have to lead healthy lives (34). SDH are dependent on multiple dimensions of the person-environment (35) and involve institutional, ecological, sociocultural, and economic levels (35, 36).

Social Accountability International (SA8000) was developed in 1997 as a best practice labor standard. SA8000 labor standards are derived from the ILO, the Universal Declaration of Human Rights (UDHR), and other international conventions. The UDHR was adopted by the United Nations in 1948 and covers the five fundamental rights of civil, political, economic, social, and cultural. International Covenants and thematic conventions include the International Covenant on Civil and Political Rights (ICCPR) and the International Covenant on Economics, Social, and Cultural Rights (ICESCR), United Nations Convention on the Rights of the Child (CRC) and the Convention on the Elimination of All Forms of Discrimination against Women (CEDAW). Key ILO conventions that underpin SA8000 include Forced Labor, Child Labor, Freedom of Association, Discrimination, Wages, Working Hours, Health and Safety, and Home workers. Countries, like Mexico, that have ratified ILO conventions must integrate labor standards into their national labor laws. SA8000 supports the operationalization of Quazi and O’Brien’s (18) model and sets a minimum standard for the legal and ethical obligations that a corporation must meet to be certified as socially accountable in the global market place.

Mexican agribusiness owners and managers are sensitive to trends and demands of the international market, however for their modern view of CSR to have an impact on farmworkers’ health and living conditions, the foreign consumer demands must challenge their international marketing activities. This agrees with the consumer-driven corporate responsibility (CDCR) model proposed by Claydon (37) that the model of CDCR proposes “that in order to remain profitable, consumer demands for CSR must be met. As a result, the corporation not only remains profitable but also engages in socially and environmentally responsible behavior, obtains a higher reputation and esteem in the public sphere due to the adoption of CSR, subsequently expands the scope of its customer base that contains more consumers who demand CSR, and hence adopts CSR that attracts more customers making them more profitable and so it continues.”

### Implications for Future Research and Policy

In Mexico, CSR research has been applied to foreign subsidiaries of multinational corporations opposed to large national companies, like Mexican agribusiness (38). Empirical CSR inquiry in Mexico has yet to measure actual socially responsible activities and performance and is limited to environmental compliance and general attitudes about CSR among Mexican corporations (39). Low/middle income country solutions to monitoring impacts of CSR are also missing (40). Growing networks of international non-governmental organizations have organized to assess, monitor, and, for some, certify CSR in worker health and safety. They set global CSR standards in line with international labor and human rights ratifications. Yet, several limitations of commercial social auditing have arisen. Auditors are expensive and lack the time, cultural, and linguistic ability to effectively audit the company from the perspective of workers, community, and health agencies affected by the existence of the corporation. Methods to assess and monitor methods focused at the local level, with existing public–private and academic partnerships, are thought to contribute to the sustained monitoring effects of CSR (41, 42). Here we juxtapose notions of CSR among agribusiness owners and the living and working conditions among their employees. Therefore a voluntary and multidisciplinary, collaborative approach to the development, implementation, and monitoring of CSR is thus required (10, 41).

The role of the organizational climate in the workplace as a moderator for employee well-being has been overlooked in workplace wellness research (43). Empirical inquiry is focused on behavior change at the individual worker level and less on social structure interventions focused on the work environment (36, 43, 44). The physical and social environments in which migrant farmworkers labor and contribute, separately and jointly, to worker well-being are even less studied and provide an opportunity to challenge the existing workplace wellness research paradigm (45).

## Conclusion

In order to understand the relationship between CSR, poverty reduction, health, and nutritional status of migrant farmworkers, conceptual and interdisciplinary social justice frameworks are necessary. According to the views and experiences of the business participants interviewed and the Quazi and O’Brien model, they understand and practice CSR from wide view and modern view; however, that is not reflected in the lives, living conditions, and physical health of their workforce. Yet, it is also clear that this concept is definitely within their frame of reference for the future. The combination of strengthening this framework at the local level, embracing a locally formed certification model and building governmental incentives could all be strategies to increase a corporate socially responsible model for migrant farmworkers in northern Mexico.

## Study Limitations

One of the main limitations of our study is the small number of owners/managers interviewed. However, there are two sample characteristics that should be accounted for: (1) interviews were obtained from owners/managers identified by the association of producers (Fundación PRODUCE) and the governmental program that oversees the migrant farmworkers (PRONJAG, the National Program with Agricultural Laborers, under the Ministry of Social Development or SEDESOL) as those largest agribusiness in the region and (2) sample of interviewed agribusiness owners were table grape farm owners and vegetable farm owners, whose differences in living conditions of farmworkers are well documented (4) and are related to requirements of certification for good agricultural practices.

## Author Contributions

All authors contributed to the research design, as well as to the acquisition, analysis, and interpretation of the data. All authors reviewed and contributed to the first draft of the paper and approved the final version for submission. All authors agreed to be accountable for all aspects of the work in ensuring that questions related to the accuracy or integrity of any part of the work are appropriately investigated and resolved.

## Conflict of Interest Statement

The authors declare that the research was conducted in the absence of any commercial or financial relationships that could be construed as a potential conflict of interest.
